# Ground water sensitivity to climate variability in the white Bandama basin, Ivory Coast

**DOI:** 10.1186/2193-1801-3-226

**Published:** 2014-05-05

**Authors:** Etienne Kouakou, Brama Koné, Alexis N’Go, Guéladio Cissé, Chinwe Ifejika Speranza, Issiaka Savané

**Affiliations:** Environnemental Sciences and Management Unit, Université Nangui Abrogoua (UNA), Abidjan, Côte d’Ivoire; Environment and Health Department, Centre Suisse de Recherches Scientifiques en Côte d’Ivoire (CSRS), Kragujevac, Côte d’Ivoire; Université Peleforo Gon Coulibaly of Korhogo, Kragujevac, Côte d’Ivoire; Ecosystem Health Science Unit, Department of Epidemiology and Public Health, Swiss Tropical and Public Health Institute (Swiss_TPH), Basel, Switzerland; Centre for Development and Environment (CDE), University of Bern, Bern, Switzerland

## Introduction

Precipitation is one of the major factors in the water balance of a catchment and as such the water cycle is most sensitive to changes in rainfall (Petheram et al. 2002). In sub-humid, semi-arid and arid regions, evapotranspiration is the second largest component of the water balance, and is affected not only by the availability of water but also by any change in other aspects of climate (e.g. temperature) (Russell et al. 2010). Some of the most useful variables for monitoring the water cycle are river flows, ocean water level, rainfall, air and water temperatures, melting of ice cap and groundwater static level (Ouarda et al. 1999; Ardoin-Bardin 2004; Gachon et al. 2007).

Analyses of hydrometric and rainfall data in Africa have demonstrated that climate is changing (Ardoin-Bardin 2004; Kouakou et al. 2007; Richard & Parker 2009). This change is characterized by a dry period which began in the 1970s and continues until the present day. A decrease was observed in rainfall intensity (Houndénou and Hernandez 1998; Mowor et al. 2009), number of rainy days (Paturel et al. 1997; Servat et al. 1999), annual rainfall (Mistry & Conway 2003; Schreck & Semazzi 2004; Black 2005; Kebede et al. 2006), and consequently river flows (Kebede et al. 2006; Mc Mahon et al. 2007; Mahé 2009) and lakes levels (Olago et al. 2009).

In western Ivory Coast, the impact of climate variability on groundwater resources in the Man area, shows a significant decrease in rainfall and river flow (Savane et al. 2001; Saley 2003). High rainfall variability with an overall rainfall deficit has led to a significant reduction in runoff of about 49% and 27% in the N'Zi and the N'Zo sub-basins of Bandama basin respectively (Goula et al. 2006, Kanohin et al. 2009). On the transborder Comoe basin, a rainfall reduction of 14% to 31%, resulted in a reduction in flows varying from 44% to 54% (Kouakou et al. 2007). Each of these studies highlights the impacts of climate variability on groundwater resources through the analysis of rainfall, river flows and the evaluation of groundwater recharge. However, these studies do not take into account groundwater level measurements. Yet the direct contact of the water table with the ground surface, unconfined aquifers, especially surficial and shallow aquifers, is particularly sensitive to changes in rainfall variability and climatic conditions (Healy & Cook 2002; Sophocleous 2002; Lee et al. 2006). Moreover, little research has been done regarding the measurement of groundwater level (Mahé et al. 2000), although groundwater water static level can serve as a variable to assess the impact of climate variability on groundwater (Ouarda et al. 1999; Jarkko & Bjorn 2010).

This paper thus aims to analyze changes in rainfall and temperature and their impact on groundwater. In addition, to the usual methods of outflow analysis and estimation of the recharge (Assani 1999; Savané et al. 2001; Kanohin 2009), this work takes into account the dynamic fluctuations of groundwater levels of boreholes in the study area.

## Methodology

### Study area

**Figure 1:**
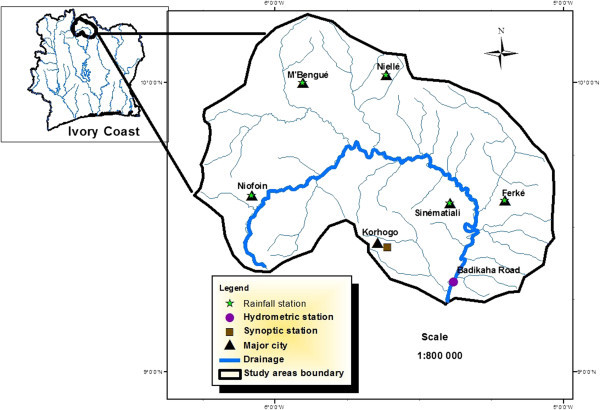
**The White Bandama Basin (source, CCT/BNETD).**

**Figure 2:**
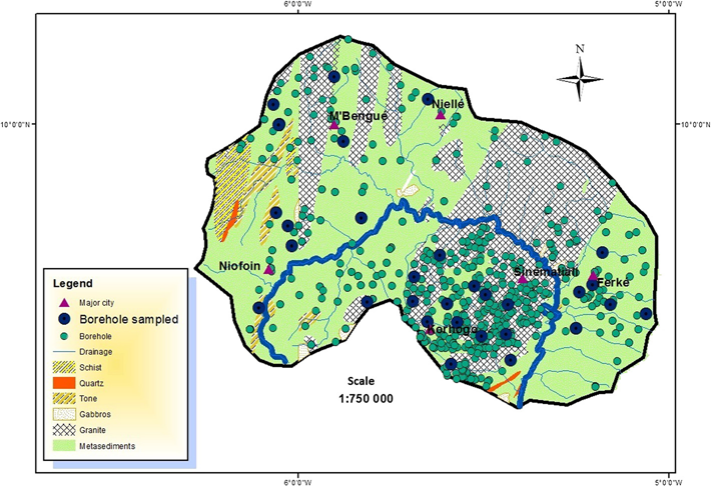
**30 boreholes sampled distribution and geological map of the basin (Laurent et al.** 1995 **).**

The White Bandama Basin (WBB) is located in northern Ivory Coast, between latitudes 9°22' and 10°26' north and between longitudes 5°00 and 6°30 west (Figure 1).With a surface area of approximately 10.050 km^2^, it is drained by a significant hydrographic network of approximately 222 km. The altitude ranges from 200 to 300 meters. The vegetation consists primarily of clear forest and savanna. As described in previous studies (Lemoine 1998; Kouamelan 1996) the lithology (Figure 2) is characterized by (1) Birimian formations that are volcanic, volcanogenic and sedimentary formations and (2) granitoid Eburnean comprising granitic solid masses in which several generations of granites are distinguished. Alterites and fracture aquifers provide a year round water supply linked to the underground grid of fractures (Sawadogo 1984; Biémi 1992). Annual rainfall ranges from 1,000 mm to 1,200 mm whith average annual temperature of 27°C. Ivory Coast in general and the study area in particular are subject to the effect of two different air masses, the Harmattan and the African monsoon:

-  the Harmattan, which is a northern trade wind brings from the North-East, dry, hot air that is often loaded with fine dust. This air mass invades the study area for several weeks from December to February.
-  the African monsoon, a humid air mass from the Atlantic Ocean. This air mass extends northwards during the months of July to September.

The climatic regime is sudanian with two climatic seasons: a dry season from October to April when rainfall is less than 100 mm and a rainy season from May to September with rainfall exceeding 100 mm. The months of December, January and February are the driest months while July, August and September are the wettest with an average monthly rainfall over 200 mm. This climate regime strongly influences the activity of 53% of the population that depend mainly on extensive agriculture. For the rest of the population, 11% are in the secondary sector (factories, construction and other industries) and 36% in the tertiary sector (trade, services, transport and administration). There are five (5) rainfall stations and one synoptic station in the basin (Figure 1).

### Data

We used the following data, drawing on the methodology adopted by Kanohin et al. (2009):

-  Mean monthly rainfall from stations of M’Bengué, Niellé and Sinématiali (1976 to 2000), Niofoin (1971 to 2000) and Ferké (1940 to 2001) and from the synoptic station of Korhogo (1944 to 2001);
-  Monthly average of maximum and minimum temperatures (1972 to 2000) from the synoptic station of Korhogo. These data were collected by SODEXAM (Société d'Exploitation et de Développement Aéroportuaire Aéronautique et Météorologique);
-  Daily river flows of White Bandama at Badikaha road station from 1981 to 1996;
-  and the characteristic of 832 boreholes (drilled between 1960 and 1996), provided by the Hydrology Service of the Ministry of Water and Forests, were used respectively for the analysis of the groundwater recharge and the variation in groundwater water static level. The measurement of thirty boreholes drilled in 1994 and spatially well distributed (Figure 2) were performed at the end of a rainy season (November 2009) and at the beginning of the dry season (May 2010). To analyse variation, the mean static levels of these 30 boreholes were compared to their original static levels. Static level measurements were made when the drillings were not pumped.

These data on rainfall, temperature and boreholes were the only useable available data. There was no missing data in the collected rainfall data. For rivers flows, missing data were corrected by linear interpolation according to the criteria described by Ardoin-Bardin (2004).

## Methods

To characterize the sensitivity of the basin’s groundwater to rainfall and temperature variability, the temporal dynamics of groundwater levels and water mobilized by the aquifer were analysed. In the following, the steps are presented.

### Break detection

Mean monthly and annual values of all the rainfall stations over the period 1944–2000 were calculated for the basin. This analysis provides information on the rainfall patterns of the basin and changes over time.

To detect breaks ( ruptures) in rainfall patterns, the segmentation procedure used by Hubert et al. (1998) and the Pettitt-test (Pettitt 1979) were applied to the annual rainfall in the basin over the period 1944–2000. The Pettitt-test is a non-parametric test based on the Mann–Whitney test. The existence of abrupt changes of some statistical parameters of hydrological series, especially their average is a possible cause of discontinuity in the series. Series of annual precipitation were checked with the segmentation process. This test allows an optimal partition of a time series into as many subsets as possible. The best segmentation is that which minimizes the squared difference between it and the series. The Scheffé test uses the notion of contrast to test if a candidate segmentation is to optimality or insignificant, verifying that the difference between all adjacent local averages taken in pairs is significant (Hubert et al. 1998). This is a test for which a significance level must be defined. Otherwise the segmentation process would continue until the segmentation of a set of n elements into n segments. The the segmentation process has the advantage of being able to search multiple changes in average of an hydrometeorological series. It is considered a stationarity test and the null hypothesis of this test is that "the series studied is stationary" (Hubert et al. 1998). This Pettitt-test has been used in various studies for this purpose(cf. Kanohin et al. 2009; Assani 1999).

### Rainfall variability analysis and reduced centered index

The reduced centred index (rainfall index) is the ratio of the deviation from the interannual mean to the standard deviation of annual rainfall. It allows to observe interannual variability and periods of deficits and excess in a rainfall series. This index was calculated for the period 1944–2000 using the formula by Nicholson et al. (1983) (Equation 1).1

where *I*_*p*_: Rainfall index; *X*_*i*_ (mm): Total rainfall per station per year i; *X*_*m*_ (mm): Mean annual rainfall per station during the study period (1944–2001) and σi: standard deviation of annual rainfall from 1944 to 2001.

### Hanning pass-low filter of 2^nd^ order and rupture detection

Following Tyson et al. (1975) (referenced in Assani 1999 and Kanohin et al. 2009), we used a moving average, also known as Hanning 2^nd^ order low-pass filter to eliminate seasonal variation in the time series. The total annual rainfall from the Korhogo station were weighted according to Assani (1999):2

for 3 ≤ *t* ≤ (*n* - 2)

where X_(t-2)_, X_(t-1)_: the total rainfall observed in the first two terms preceding the term X_(t)_; X_(t+1)_, X_(t+2)_: are the total rainfall observed for the first two terms after the term X_(t)_.

The total rainfall weighted for the two first terms (X_1_, X_2_) and the two last terms (X_(n-1)_, X_n_) under the series are calculated using the following equations (n: size of the series):3456

### Interannual trend of monthly mean rainfall and temperature

To highlight current trends in rainfall over the basin, interannual variations of monthly mean rainfall were analyzed from 1971 to 2001. These changes are displayed in histograms. Changes in maximum and minimum temperatures average from 1972 to 2000 were also analyzed only for the synoptic station of Korhogo.

### Dynamics of groundwater resources over time

Analyses were performed of the two climate seasons of the basin (dry and wet). The static levels of 832 boreholes (drilled between 1960 and 1996) measured during their establishment were analyzed in order to characterise their distribution. Subsequently, the average of static level messures performed on 30 boreholes in 2009 and 2010 following the two seasons were compared to one other and then compared to the their static level at their establishment according Mahé et al. (2000). The Student's t-Test was applied to these series. Linear correlation between river flow and rainfall, between borehole static level and rainfall and between borehole static level and years was established.

### Recession coefficient and water mobilized by the aquifer

We used the recession coefficient of Maillet (k) improved by dichotomous resolution and the volumes of water mobilized (Vm) by aquifer, presented by Savane et al. (2001). The equation of the recession coefficient is described by:7

where:

*Q*_*t*_: Flow at the time t, *Q*_0_: initial flow (discharge at the beginning of drying) and recession coefficient k of Maillet obtained by solving the equation (8) below:8

with:

V = the volume sold at any time (m^3^).

Integrating equation (9) on the interval [0, + α] gives the volume of water mobilized by all aquifers in the watershed:9

The water balance was established using the method of Thornthwaithe (Thornthwaite et Matter 1957):

## Results

### Breakdown detection

The segmentation procedure of Hubert et al. (1998) applied to the rainfall partterns in the basin highlights a break in stationarity in 1970. These segmentations were obtained at the 0.05 level of significance of the Scheffe test. This break is confirmed by Pettitt test.

### Changes in rainfall

**Figure 3:**
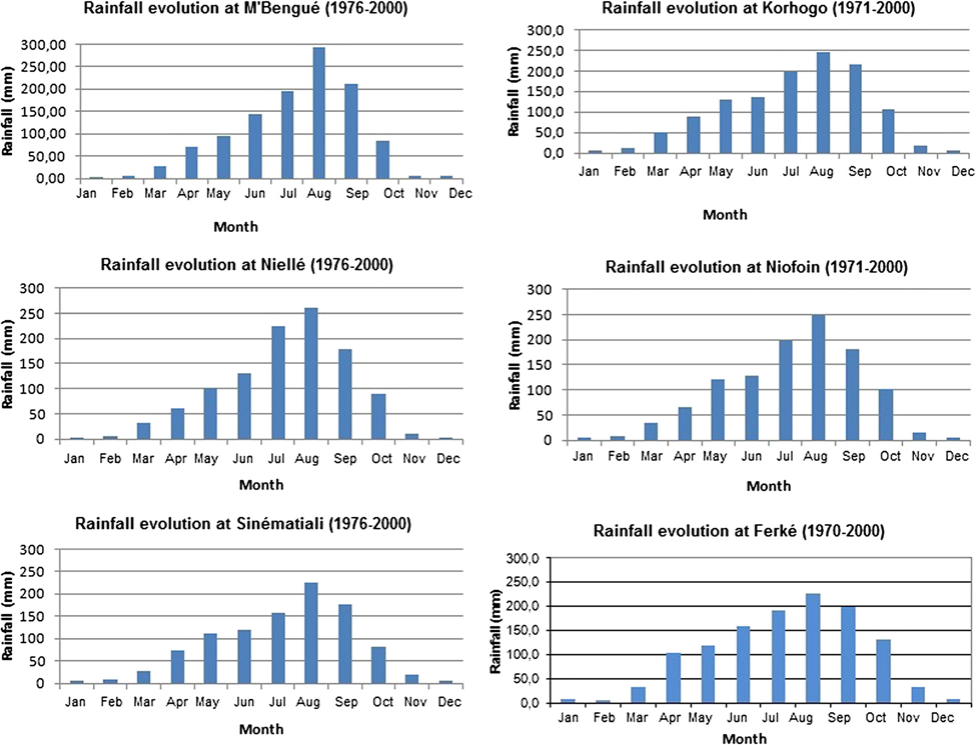
**Rainfall distribution in the rainfall stations and the synoptic station of the basin.**

The analysis of total annual rainfall shows that average rainfall amounts vary between 0 and 300 mm (Figure 3). All graphs in this figure have the same distribution. An inceasing phase from April to August and a decreasing phase from August to April. From October to April, rainfall is generally less than 100 mm, this correspond to the dry seasons. And from May to September, we have the rany season. August is the wettest month of the year, followed by the months of July and September, while the months of December, January and February are the driest.

**Figure 4:**
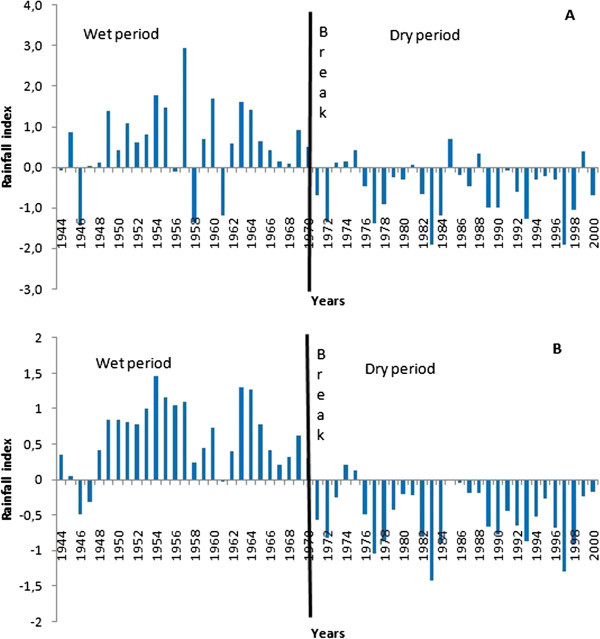
**Change in rainfall indices (A) and reduced centered index and (B) from 1944 to 2000 on the White Bandama Basin.**

The interannual fluctuations in rainfall on the White Bandama Basin is characterized by a wet period from 1944 to 1970 followed by a dry period from 1971 to 2000 (Figure 4A). The Hanning low-pass filter highlights more the different periods of rainfall deficits and surpluses (Figure 4B). The dry period has some remarkable years: 1977, 1983 and 1997 marked by severe drought. The mean annual rainfall during periods before and after this break (1970) are 1383 mm and 1213 mm respectively, showing an annual rainfall decrease of 12% between the two periods.

**Figure 5:**
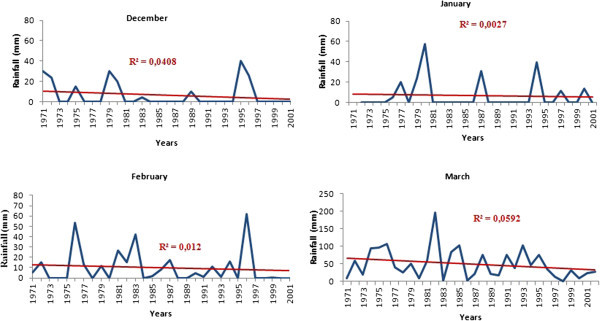
**Annual distribution of driest months rainfall from 1971 to 2001 at Korhogo station.**

**Figure 6:**
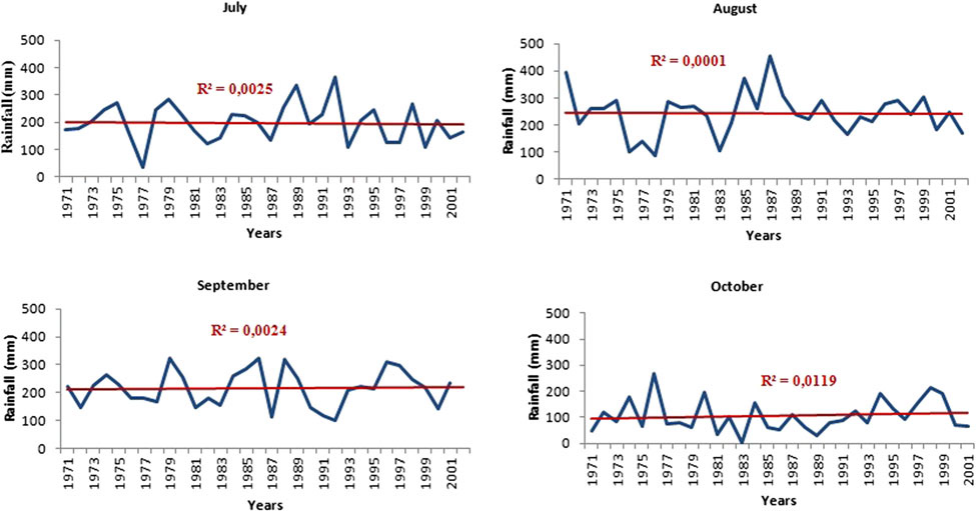
**Annual distribution of wettest months rainfall from 1971 to 2001 at Korhogo station.**

The analysis of inter-annual monthly rainfall of driest and wettest months from 1971 to 2001 shows a marked decline in rainfall for all months except September and October. Figure 5 shows the trend in inter-annual monthly average rainfall of the driest months in the basin, which are all decreasing. It is the same for the wettest months except for September and October (Figure 6) experiencing an increase in rainfall. For the months of July and August, there was a very slight decline.

### Changes in temperature

**Figure 7:**
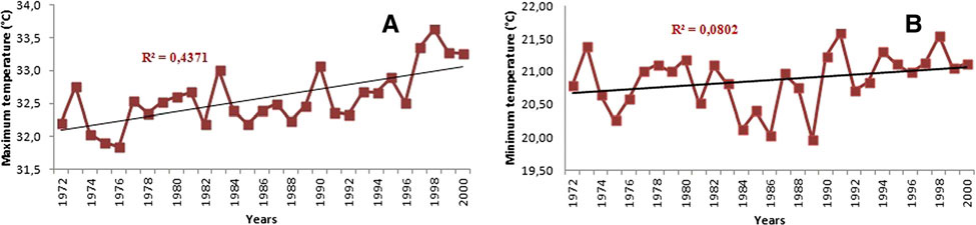
**Distribution of the average of annual maximum (A) and minimum (B) temperature from 1972 to 2000 at Korhogo station.**

As the rainfall decreases, there is an increase in mean annual maximum temperature over the period 1972 to 2000 with peaks occurring on average every six years (Figure 7A). These peaks in the years 1973, 1980, 1987, 1991 and 1998 correspond to years of drought in Figure 4b (after the the break). The peaks have increased with monthly mean intensities rising above 32.5°C, over time. For minimum temperature (Figure 7B), there is an increase from ~20°C in 1989 to 21.6°C in 1991 and since then, minimum temperatures are around 21°C, an increase of about 1°C. The picks observed for the maximum temperature are also observed for minimum temperature of the same years.

### Groundwater sensitivity to rainfall and temperature change

#### Change in water static level of boreholes

**Figure 8:**
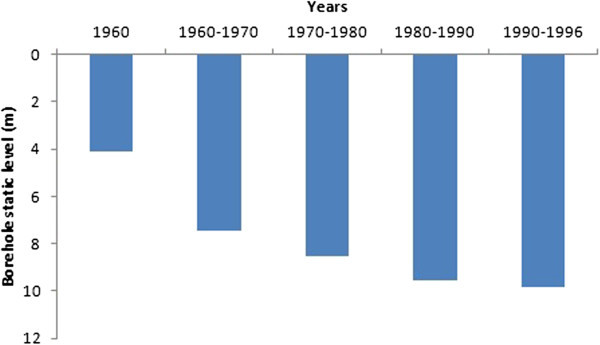
**Changes in the 832 boreholes statics levels from 1960 to 1996 in the WBB.**

**Figure 9:**
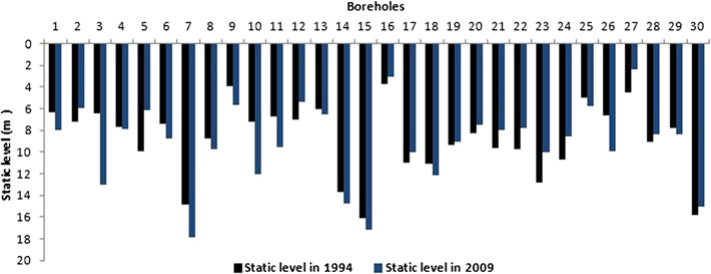
**30 boreholes mean static level variation between 1994 and 2009 on the White Bandama Basin.**

The analysis of the values of static levels of 832 selected boreholes over the period 1960–1996, shows a decrease (Figure 8). The mean static level declines from 4.42 m before 1960 to 9.86 m during 1990–1996, corresponding to a drop of about 5.44 m over the period. The t-test applied to this series shows a significant difference. Comparison of static level measurements made in 2009 for 30 boreholes to initial static levels showed a significant decrease in the static level (Figure 9).

The mean water static level in 30 boreholes dropped from 8.89 m in 1994 to 9.29 m in 2009 representing a decrease of about 0.4 m. Although this decline was not statistically significant, we note that over half of these boreholes have their static levels lower than 1994 levels. The maximum static level (the lowest) also decreased from 16.1 m to 17.85 m between the two periods. Moreover, there is a significant interseasonal variation marked by a mean difference of static level of 3.58 m between the dry season and the rainy season.

**Figure 10:**
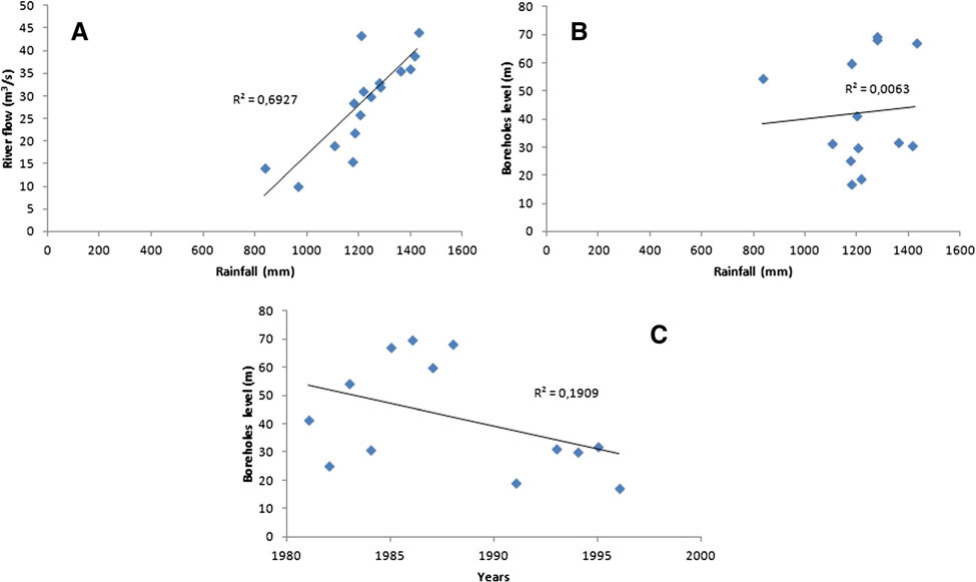
**Linear correlation between rainfall and river flow (A) and between borehole static level and rainfall (B) and between borehole static level and years (C).**

There is an important increasing linear correlation between rainfall and river flow with a correlation coefficient of R^2^ = 0.69 (Figure 10A). When rainfall increases, river flow also increases. We also observe low growing linear correlation (R^2^ = 0.009) between the static level of boreholes and rainfall (Figure 10B). This means that when the rainfall increases, the static level also increases. In addition, there is also a weak decreasing correlation between the boreholes static level and years with R^2^ = 0.19 (Figure 10C).

#### Recession coefficient and water mobilized by WBB’s aquifers

**Figure 11:**
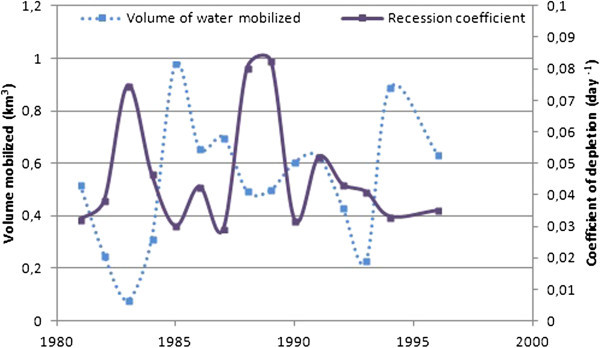
**Recession coefficient and water volume mobilized by aquifers in Bandama Basin between 1981 and 1996.**

Figure 11 shows the distribution of the recession coefficient (drying up) and the volume of water mobilized in the basin between 1981 and 1996.

Recession coefficients varied from 0.029 day^-1^ to 0.082 day^-1^ between 1981 and 1996 with an average of 0.047 day^-1^. The highest values of this coefficient were between the periods 1982–1984 and 1987–1990. These periods correspond to observed periods of severe drought.

During these periods, the recession coefficient increased greatly, reflecting an inability of aquifers to properly recharge, particularly in 1983. The recession coefficient moved in the opposite direction to the volume of water mobilized by the aquifers annually. This means that, when the recession coefficient is low, the volume of water mobilized by the aquifer is high. This volume varied from 0.076 km^3^ per year in 1983 to 0.98 km^3^ per year in 1985. Since 1985, the volume of water mobilized has remained low, particularly during 1993 characterized by the lowest volume. The recession coefficient is still, in general, quite high in comparison to the volume of water mobilized. This reflects a general downward trend in the volume of water mobilized between 1981 and 1996.

### Water balance

Due to lack of temperature data for the period before 1970, we analyze water balance for the period after the break (1972–2000) and estimated recharge from the effective infiltration. The Table 1 below summarizes the key terms of the balance sheet from 1972 to 2000. It is observed that the ETP is very important in the basin, accounting for approximately 71% of the rainfall.

**Table 1:** **Water balance from 1972 to 2000**

Rainfall (mm)	1213,4
Evapotranspiration (ETP) (mm)	861,5
Infiltration (mm)	205,34
Volume of water mobilized (km^3^)	2,06

## Discussion

The decrease observed in rainfall during the period 1971–2000 compared to the period 1944–1970 fits well the climatic context in Ivory Coast (Saley 2003; Kanohin et al. 2009, Soro et al. 2011). These authors observed lower rainfall of the rainiest months in these regions. On the transboundary basin of Comoé, a decline of about 13% in rainfall for stations located on the Ivorian basin has been noted (Kouakou et al. 2007). This high rainfall variability in Ivory Coast also fits the West Africa subregion climate, which experienced a decline in rainfall since the late 1960s and early 1970s (Hubert & Carbonnel 1987; Moron 1994; Paturel et al. 1995; Paturel et al. 1997; Servat et al*.*^1999^; Conway et al. 2009; Soro et al. 2011). Although apparently not statistically significant, the overall trend is downward for the driest months and rising for the rainiest months. If the observed inter-annual decrease in rainfall continues, the basin could be subjected to climatic conditions similar to Sahelian countries. The increase in rainfall in September and October shows a concentration of annual rainfall over a short period. Unlike rainfall, minimum temperature increased to about 1°C with a return period of approximately eight (8) years for maximum temperature. These peaks sometimes, correspond to periods of severe drought (1983, 1997, and 1990) and contribute to increaseing the pressure on the basin’s water resources. This is indeed observed through high-level static variation between the rainy season and the dry season.

Figure 10 (A, B and C), shows that rainfall variation influences river flow and borehole static level. Also, borehole static level seems to be influenced by time (years). The weak correlation between borehole static level and rainfall could be explained by the fact that the change does not occur immediately after a rainfall event. The poor correlation between the years and the borehole static level from 1981 to 1996 could mean that, could mean that other factors influence the static level. The basin is a fractured nappe (Jourda et al. 2006). Although some fractures are interconnected, they are not evenly distributed across the basin. Also, the population density is quite low in most major cities of the study area. It’s about 14 inhabitants/km^2^, 15 inhabitants/km^2^ and 32 inhabitants/km^2^ in Ferké, M'Bengué and Korhogo, respectively. Moreover, boreholes are only used for drinking purpose and not for agricultural activities that may need much more water. For all these reasons, we agree that even if anthropogenic ground water abstraction may exist, that can not caused a general decrease of static levels in all parts of the basin. A general decrease, as observed, may be mainly linked to a factor that affects the entire basin, like rainfall (mean rainfall). Hence, the long period of deficit could play an important role in lowering the water static level in the basin. This has been demonstrated by Mahé et al. (2000) who using the method of coefficients, have analyzed the differences between rainfall, streamflow and piezometric levels of 27 boreholes in the Bani basin of Mali. Their results show a decreased static level that correlated with rainfall. Adja et al. (2009), analyzing satellite images (from 1999 to 2000) of the WBB, showed that three months after the rainy season, rivers lose about 20% of their water due to evaporation. This rate increases to 66%, four months after the rainy season. The rivers of the study area experienced a rapid reduction between 50 and 60 days (Jourda et al. 2006). This may explain the high rate of recession coefficient in the basin between 1981 and 1996. Indeed, there are complex relationships between rivers and groundwater that vary over the seasons. The recession coefficient is proportional to the rate of drying up and increases in dry periods. This relationship between rainfall and river flow, is confirmed by our results especially through the water balance.

Generally, success in natural resources management depends on precise diagnosis of the state and distribution of the resources. It also depends on a clear identification of active agents and driving forces that sustain negative trends in resource availability or access. Population pressure, political and socio-economic factors, which are not the focus of this study, can also influence water resources conditions. Our results, derived from the analysis of rainfall and temperature shows that these climatic factors influence the state of water resources in the basin.

Difficulties related to the availability, accessibility and quality of data was a limitation for this study. Also, climate-related impacts on groundwater are difficult to establish over short-timescales and long-term data are usually lacking (Bovolo and Parkin 2009).

## Conclusion

Our aim was to analyze the impact of climate variability on groundwater resources of the White Bandama Basin. The statistical methods used break detection procedures to analyse rainfall variability, the temporal dynamics of groundwater levels and water mobilized by the aquifers.

The results showed a decrease in rainfall of the basin since a break in 1970 while temperatures are increasing. During the period 1971 to 2001, wettest and driest months except September and October experinced a drop in rainfall. There is also a decline in the water static level that cannot be due only to the effects of temperature and rainfall. Also, the high rate of recession coefficient influences the groundwater recharge causing a decrease in water mobilized by aquifers.

With regard to climate variability and it impacts on water resources, it is important to create conditions for sustainable management of water resources for future challenges.

To better assess the contribution of rainfall and temperature to changes in groundwater resources, it is necessary that future studies also analyse other possible influence factors such as the role of the substratum, the population and socio-economic activities conducted in the basin.
